# Betulinic Acid Ameliorates the Severity of Acute Pancreatitis via Inhibition of the NF-κB Signaling Pathway in Mice

**DOI:** 10.3390/ijms22136871

**Published:** 2021-06-26

**Authors:** Ziqi Zhou, Ji-Won Choi, Joon Yeon Shin, Dong-Uk Kim, Bitna Kweon, Hyuncheol Oh, Youn-Chul Kim, Ho-Joon Song, Gi-Sang Bae, Sung-Joo Park

**Affiliations:** 1 Department of Herbology, School of Korean Medicine, Wonkwang University, 460 Iksandaero, Iksan 54538, Jeonbuk, Korea; zzq0325@naver.com (Z.Z.); agraesb1@naver.com (J.-W.C.); joonyeon3@naver.com (J.Y.S.); kbn306@naver.com (B.K.); songhj@wku.ac.kr (H.-J.S.); 2 Hanbang Cardio-Renal Syndrome Research Center, School of Korean Medicine, Wonkwang University, 460 Iksandaero, Iksan 54538, Jeonbuk, Korea; ckck202@naver.com; 3 Institute of Pharmaceutical Research and Development, College of Pharmacy, Wonkwang University, 460 Iksandaero, Iksan 54538, Jeonbuk, Korea; hoh@wonkwang.ac.kr (H.O.); yckim@wku.ac.kr (Y.-C.K.); 4 Department of Pharmacology, School of Korean Medicine, Wonkwang University, 460 Iksandaero, Iksan 54538, Jeonbuk, Korea; 5 Research Center of Traditional Korean Medicine, Wonkwang University, 460 Iksandaero, Iksan 54538, Jeonbuk, Korea

**Keywords:** acute pancreatitis, betulinic acid, acinar cell, inflammation, nuclear factor-kappa B

## Abstract

Acute pancreatitis (AP) is an inflammatory disorder, involving acinar cell death and the release of inflammatory cytokines. Currently, there are limited effective therapeutic agents for AP. Betulinic acid (BA) is a pentacyclic triterpenoid extracted from *Betula platyphylla* that has been shown to have anti-inflammatory effects. In this study, we aimed to investigate the effects of BA on AP and elucidate the potential underlying mechanisms. AP was induced in mice through six intraperitoneal injections of cerulein. After the last cerulein injection, the mice were sacrificed. Our results revealed that pre- and post-treatment with BA significantly reduced the severity of pancreatitis, as evidenced by a decrease in histological damage in the pancreas and lung, serum amylase and lipase activity and pancreatic myeloperoxidase activity. Furthermore, BA pretreatment reduced proinflammatory cytokine production, augmentation of chemokines, and infiltration of macrophages and neutrophils in the pancreas of AP mice. In addition, mice that were pretreated with BA showed a reduction in Iκ-Bα degradation and nuclear factor-kappa B (NF-κB) binding activity in the pancreas. Moreover, BA reduced the production of proinflammatory cytokines and NF-κB activation in pancreatic acinar cells (PACs). These findings suggest that BA may have prophylactic and therapeutic effects on AP via inhibition of the NF-κB signaling pathway.

## 1. Introduction

Acute pancreatitis (AP) is a complex disease, caused by the release of active pancreatic enzymes and the invasion of inflammatory cells [1]. The majority of patients suffer from mild or edematous AP with a low complication rate, and about 20% of patients develop severe pancreatitis accompanied by multiple organ failure [2]. Although the pathogenesis of AP remains unclear, pancreatic damage is initiated by acinar cell injury followed by the production of proinflammatory cytokines and mediators, leading to the activation of immune cells [3]. These cells escalate the inflammatory response, resulting in increased damage to the pancreas [4]. Therefore, to improve the outcome of patients with AP, it is necessary to understand the molecular mechanisms involved and develop effective therapeutic modalities.

Nuclear factor-κB (NF-κB) heterodimers consist of p50 and RelA (also known as p65) in complex with the inhibitory κ-Bα (Iκ-Bα), which sequesters them in the cytoplasm under a steady state [5]. In an inflammatory response, Iκ-Bα protein is phosphorylated via activation of the IκB kinase (IKK) complex IKKα/IKKβ, which then allows proteasome-mediated degradation of Iκ-Bα [6]. Through this process, NF-κB translocates to the nucleus and mediates the expression of several inflammatory mediators, including cytokines, chemokines, and adhesion molecules [7]. NF-κB is activated in acinar cells during the early stages of AP and is involved in the progression of inflammatory responses [8]. Strategies for NF-κB inhibition have previously been attempted in various experiments on AP [9,10]. Therefore, pharmacological inhibition of NF-κB activation would be beneficial for pancreatitis treatment.

Betulinic acid (BA), a natural pentacyclic triterpene isolated from *Betula platyphylla,* exhibits various biological activities, such as anti-inflammatory, antioxidant, antifibrotic, and anticancer [11,12,13,14,15,16]. Furthermore, it has been shown to reduce inflammatory responses in various animal models, such as those for diabetes-induced renal fibrosis [17], chemical-induced hepatic fibrosis and fatty liver [18], and chronic stress-induced depression [19]. However, the effects of BA on AP remain unknown.

In this study, we provided evidence that BA ameliorates the severity of AP in a mouse model with cerulein-induced AP and pancreatic acinar cells (PACs), in addition to elucidating the potential mechanism underlying this effect.

## 2. Results

### 2.1. Biochemical Toxicity of BA

To investigate the toxicity of BA in vivo, levels of the biochemical factors in the serum were assessed at 24 h after administration of DMSO or BA. As shown in Table 1, administration of BA at a dose of 20 mg/kg caused slight toxicity compared to the DMSO treatment, as indicated by an increase in ALT levels. Therefore, in subsequent experiments, BA was used at concentrations below 20 mg/kg.

### 2.2. Effect of BA on Pancreatic Damage in Cerulein-Induced AP

**Figure 1:**
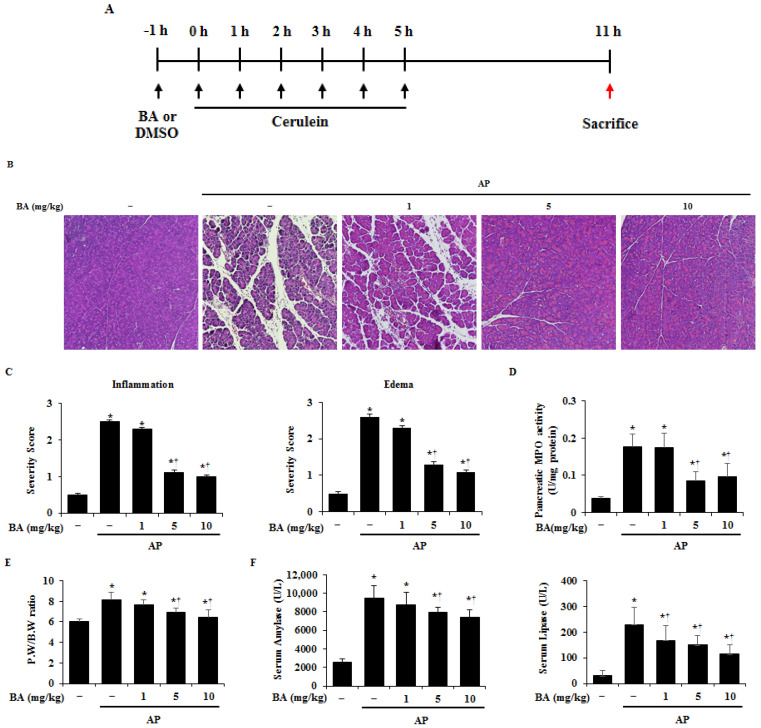
Effect of betulinic acid (BA) on pancreatic damage in cerulein-induced acute pancreatitis (AP). (**A**) Mice were pretreated with BA (1, 5, or 10 mg/kg) or dimethyl sulfoxide (DMSO) for 1 h, then cerulein (50 µg/kg) was intraperitoneally injected into the mice every hour for 6 h. The mice were then sacrificed at the end of 6 h, that is, after the last cerulein injection. (**B**) Representative hematoxylin and eosin (H&E)-stained pancreas section (200× magnification). (**C**) Histological sections of the pancreas were scored from 0 (normal) to 3 (severe) for inflammation and edema. (**D**) Pancreatic myeloperoxidase (MPO) activity. (**E**) Pancreas weight/body weight (PW/BW) ratio. (**F**) Serum levels of amylase and lipase. Data have been represented as mean ± SEM for six mice in each group. Results are representative of three experiments. * *p* < 0.05 vs. DMSO treatment alone. ^†^ *p* < 0.05 vs. cerulein treatment alone.

To investigate the effects of BA in AP, BA (1, 5, or 10 mg/kg) or DMSO was administered 1 h before the first cerulein (50 µg/kg) injection (Figure 1A). The severity of AP was assessed by examining morphological evidence of the extent of acinar cell injury, edema (PW/BW ratio), inflammatory cell infiltration, and neutrophil sequestration (MPO activity) [20]. As shown in Figure 1B–E, pretreatment with BA reduced pancreatic damage, compared to that of DMSO treatment, as evidenced by the reduced acinar cell death, pancreatic edema, inflammatory cell infiltration, and MPO activity. In addition, we examined the levels of digestive enzymes in the serum, such as amylase and lipase, which are known to increase in AP. Pretreatment with BA also reduced the elevated amylase and lipase activities in the serum (Figure 1F).

### 2.3. Effect of BA on Lung Histological Damage in Cerulein-Induced AP

**Figure 2:**
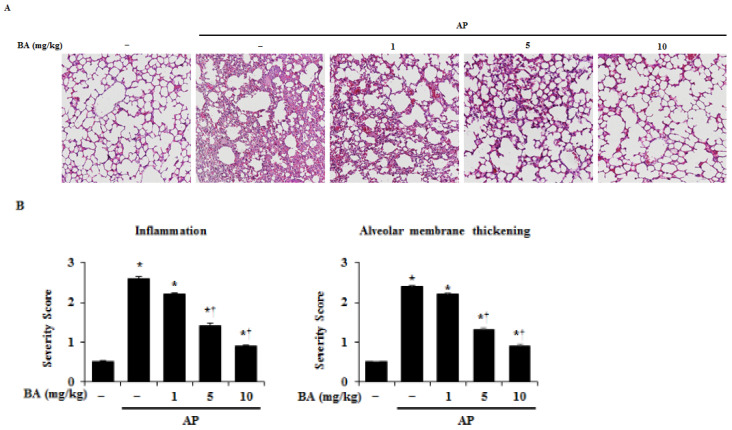
Effect of betulinic acid (BA) on lung histological damage in cerulein-induced acute pancreatitis (AP). (**A**) Representative hematoxylin and eosin (H&E)-stained lung tissue section (200× magnification). (**B**) Histological sections of lung tissue were scored from 0 (normal) to 3 (severe) for inflammation and alveolar membrane thickening. Data have been represented as mean ± SEM for six mice in each group. Results are representative of three experiments. * *p* < 0.05 vs. DMSO treatment alone. ^†^ *p* < 0.05 vs. cerulein treatment alone.

Because lung injury is a common complication of AP [21], we evaluated lung injury during AP in our models. As shown in Figure 2, administration of cerulein-induced lung injury was shown by an increase in inflammatory cell infiltration and alveolar membrane thickening. However, pretreatment with BA decreased the lung injury during AP.

### 2.4. Effect of BA on Production of Proinflammatory Cytokines

**Figure 3:**
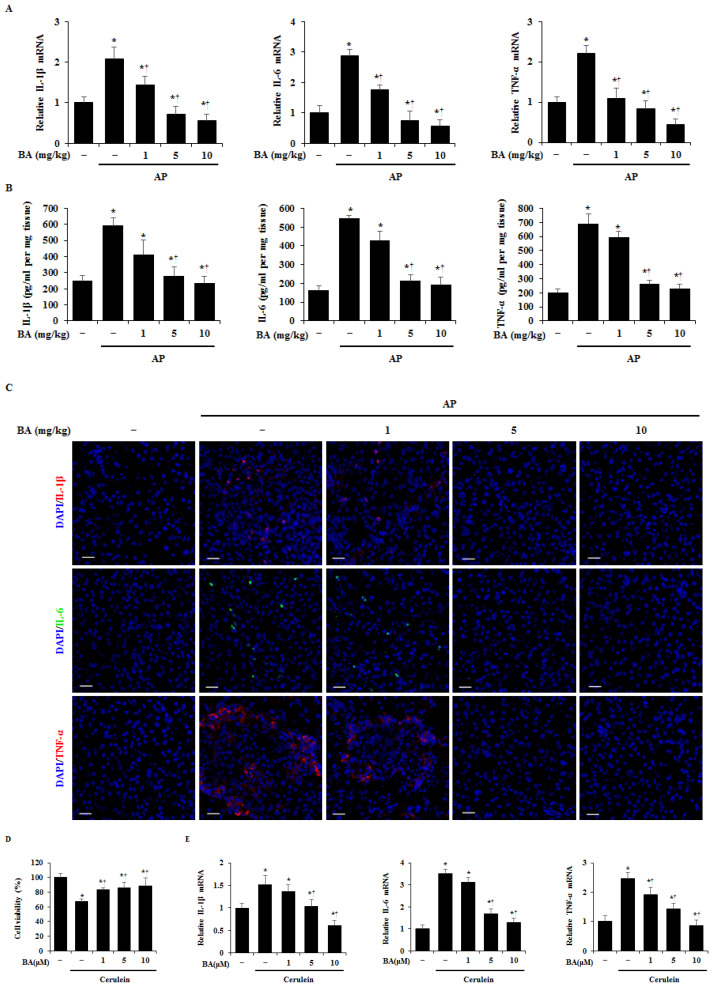
Effect of betulinic acid (BA) on production of proinflammatory cytokines. (**A**) Pancreatic mRNA levels of interleukin (IL)-1β, IL-6, and tumor necrosis factor (TNF)-α were determined using reverse transcription-quantitative polymerase chain reaction (RT-qPCR). (**B**) Pancreatic protein levels of IL-1β, IL-6, and TNF-α were measured by ELISA. (**C**) Confocal images of immunofluorescence staining of IL-1β (red), IL-6 (green), TNF-α (red), and DAPI (blue). Scale bar = 20 μm. (**D**) Pancreatic acinar cells (PACs) were pretreated with BA (10 µM) for 1 h and then stimulated with cerulein (10 nM) for 6 h. The cell viability was measured as described in the experimental protocol. (**E**) PACs were pretreated with BA for 1 h at the indicated dose, and then stimulated with cerulein (10 nM) for 24 h. mRNA levels of IL-1β, IL-6, and TNF-α in PACs were determined using RT-qPCR. Data have been represented as mean ± SEM for six mice in each group. Results are representative of three experiments. * *p* < 0.05 vs. dimethyl sulfoxide (DMSO) treatment alone. ^†^ *p* < 0.05 vs. cerulein treatment alone.

AP causes the release of proinflammatory cytokines, such as interleukin (IL)-1β, IL-6, and tumor necrosis factor (TNF)-α [22]. Therefore, we examined whether BA reduces the production of proinflammatory cytokines in the pancreas during AP. As shown in Figure 3A–C, the expression levels of IL-1β, IL-6, and TNF-α mRNAs and proteins in the pancreas were significantly increased in cerulein-induced AP, while BA administration reduced the expression of these cytokines. In addition, COX-2 is a crucial and central mediator in the development and severity of AP [23,24,25,26]. Hence, we examined the effect of BA on COX-2 in the pancreas. As shown in Supplementary Figure S1, administration of BA reduced the levels of COX-2 mRNA and protein during cerulein-induced pancreatitis. An early event of AP is cell death and proinflammatory cytokine production in acinar cells [27]. Therefore, we investigated the effect of BA on cell death and proinflammatory cytokine production using isolated PACs. BA inhibited cell death and mRNA expression of cytokines, such as IL-1β, IL-6, and TNF-α in a dose-dependent manner in PACs (Figure 3D,E).

### 2.5. Effect of BA on Macrophage and Neutrophil Infiltration into the Pancreas

**Figure 4:**
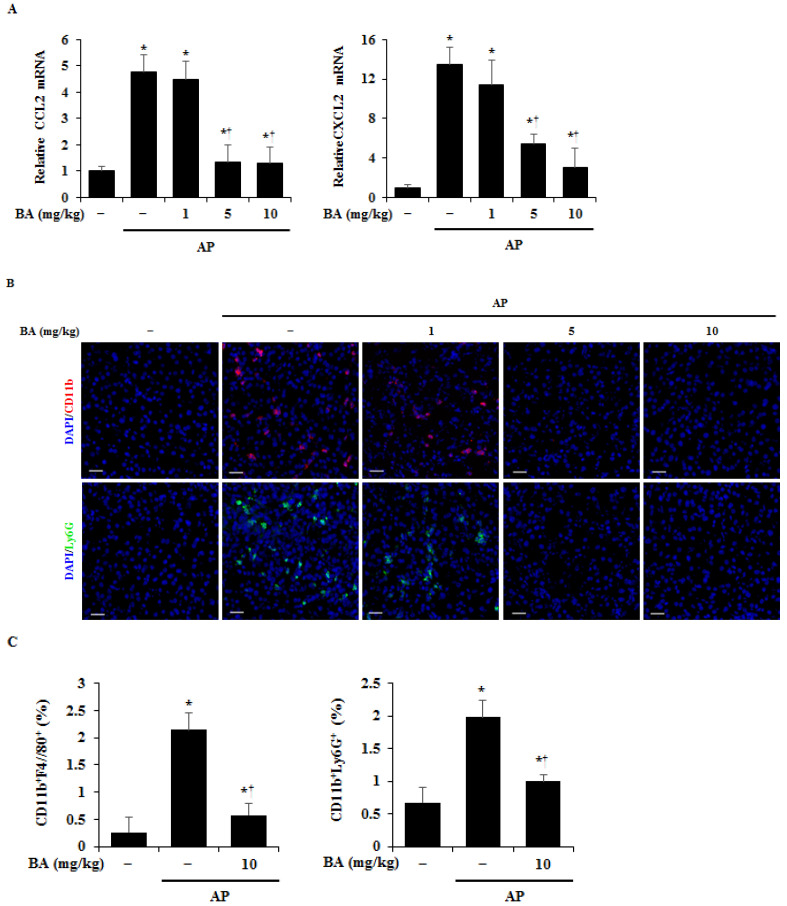
Effect of betulinic acid (BA) on the infiltration of macrophages and neutrophils. (**A**) Pancreatic mRNA levels of chemokine (CC motif) ligand 2 (CCL2) and chemokine CXC motif ligand 2 (CXCL2) were determined using reverse transcription-quantitative polymerase chain reaction (RT-qPCR). (**B**) Confocal images of immunofluorescence staining of CD11b (red), Ly6G (green), and DAPI (blue). Scale bar = 20 μm. (**C**) Flow cytometry analysis for macrophages (CD11b^+^F4/80^+^) and neutrophils (CD11b^+^Ly6G^+^). Data are represented as mean ± SEM for six mice in each group. Results are representative of three experiments. * *p* < 0.05 vs. dimethyl sulfoxide (DMSO) treatment alone. ^†^ *p* < 0.05 vs. cerulein treatment alone.

One of the major characteristics of AP is the infiltration of immune cells, such as macrophages and neutrophils, into the pancreas, with elevated inflammatory cytokines being closely related to these immune cells [28]. Therefore, to determine the effect of BA on immune cell infiltration into the pancreas we measured chemokines, such as chemokine (CC motif) ligand 2 (CCL2) and chemokine CXC motif ligand 2 (CXCL2). These chemokines in the pancreas were increased in cerulein-induced AP at the mRNA level, whereas pretreatment BA dramatically reduced in a dose-dependent manner (Figure 4A). Furthermore, we observed macrophages and neutrophils in the pancreas by immunofluorescence staining with CD11b (for macrophage) and Ly6G (for neutrophils). The number of CD11b or Ly6G positive cells were high in the AP group, however, pretreatment with BA reduced the number of CD11b or Ly6G positive cells to the level of the normal group (Figure 4B). To quantify these results in the pancreas during AP, we detected the macrophages with CD11b^+^F4/80^+^ cells and the neutrophils with CD11b^+^Ly6G^+^ cells. Double positive cells (CD11b^+^F4/80^+^ or CD11b^+^Ly6G^+^) were increased significantly in AP. However, the increase was inhibited by BA treatment, in accordance with our immunofluorescence data (Figure 4C).

### 2.6. Effect of BA on NF-κB Activation

**Figure 5:**
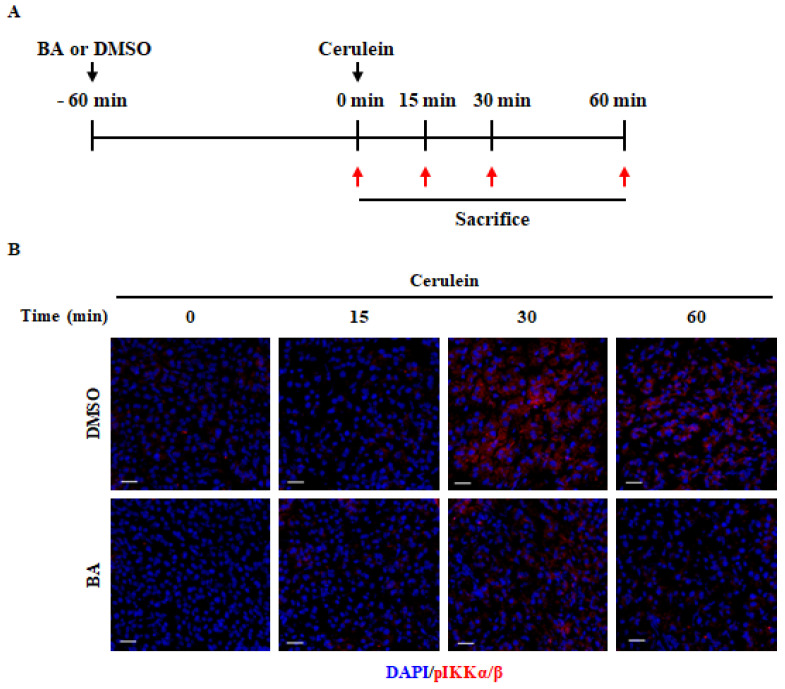

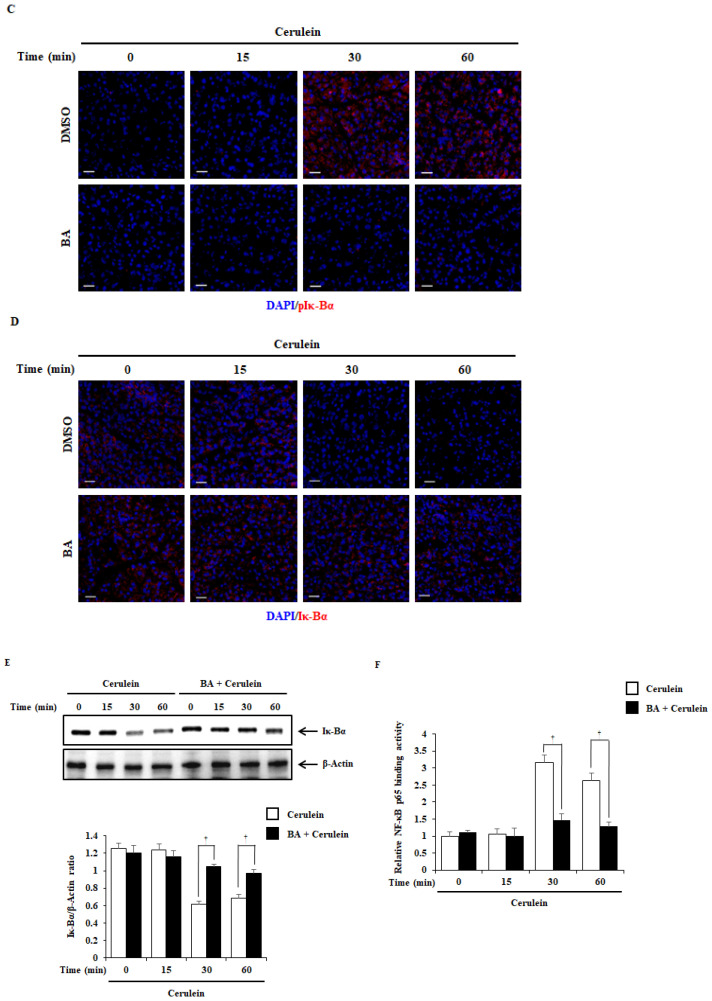

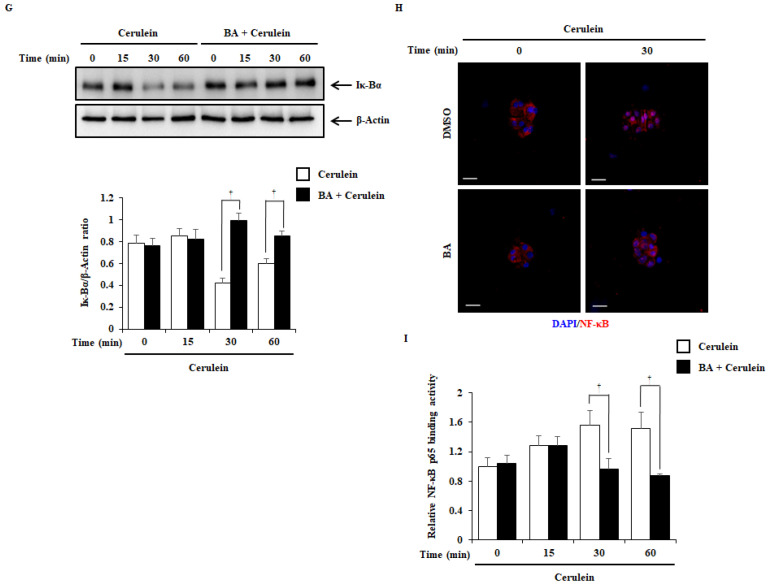
Effect of betulinic acid (BA) on nuclear factor-kappa B (NF-κB) activation. (**A**) Mice were pretreated for 1 h with BA (10 mg/kg) or dimethyl sulfoxide (DMSO). The mice were then injected with cerulein (50 µg/kg) and sacrificed at 0, 15, 30, or 60 min. Confocal images of immunofluorescence staining of (**B**) phosphorylated IκB kinase (pIKKα/β, red), (**C**) phosphorylated inhibitory κ-Bα (pIκ-Bα, red), (**D**) Iκ-Bα (red) and DAPI (blue). Scale bar = 20 μm. (**E**) Degradation of Iκ-Bα was measured using western blotting. β-Actin was used as the loading control. The western blot of Iκ-Bα was quantified by densitometry and normalized to β-Actin. (**F**) NF-κB p65 binding activity was measured in the pancreas. (**G**) Pancreatic acinar cells (PACs) were pretreated with BA (10 µM) for 1 h and then stimulated with cerulein (10 nM) for the indicated times. Degradation of Iκ-Bα was measured using western blotting. β-Actin was used as the loading control. The western blot of Iκ-Bα was quantified by densitometry and normalized to β-Actin. (**H**) Immunofluorescence staining of NF-κB p65 (red) and DAPI (blue) in PACs. Scale bar = 20 μm. (**I**) NF-κB p65 binding activity was measured in PACs. Data are represented as mean ± SEM for six mice in each group. Results are representative of three experiments. ^†^ *p* < 0.05 vs. cerulein treatment alone.

To examine the inhibitory mechanism of BA in cerulein-induced AP, we investigated the NF-κB and mitogen activated protein kinase (MAPK) signaling pathway in the pancreas, which may be involved in the regulation of cytokine production [29]. Immunofluorescence staining demonstrated that the expression of pIKKα/β and pIκ-Bα in the pancreas was significantly increased at 30 min, compared to that in the control group. However, the increase in these levels was significantly inhibited upon BA pretreatment (Figure 5B,C). Furthermore, we examined the degradation of Iκ-Bα protein and NF-κB binding activity in the pancreas. As shown in Figure 5D–F, the administration of BA inhibited the degradation of Iκ-Bα and NF-κB binding activity in the pancreas. However, the administration of BA did not inhibit the phosphorylation of P38, c-Jun *N*-terminal kinase (JNK), and extracellular signal-regulated kinase (ERK) (Supplementary Figure S2). NF-κB is activated early in acinar cells during AP [30]. Next, we examined the NF-κB signaling pathway in the isolated PACs. In accordance with the in vivo test results, BA significantly inhibited the degradation of Iκ-Bα protein, NF-κB p65 translocation into the nucleus, and NF-κB binding activity (Figure 5G–I).

### 2.7. Therapeutic Effects of BA in Cerulein-Induced AP

**Figure 6:**
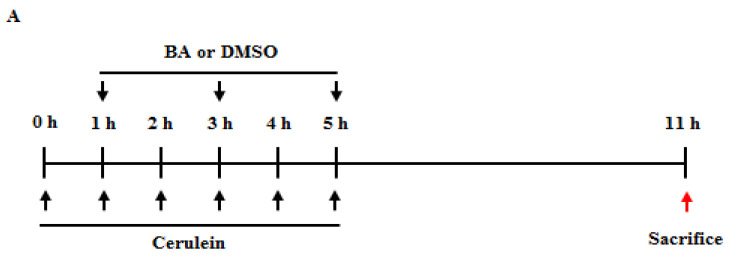

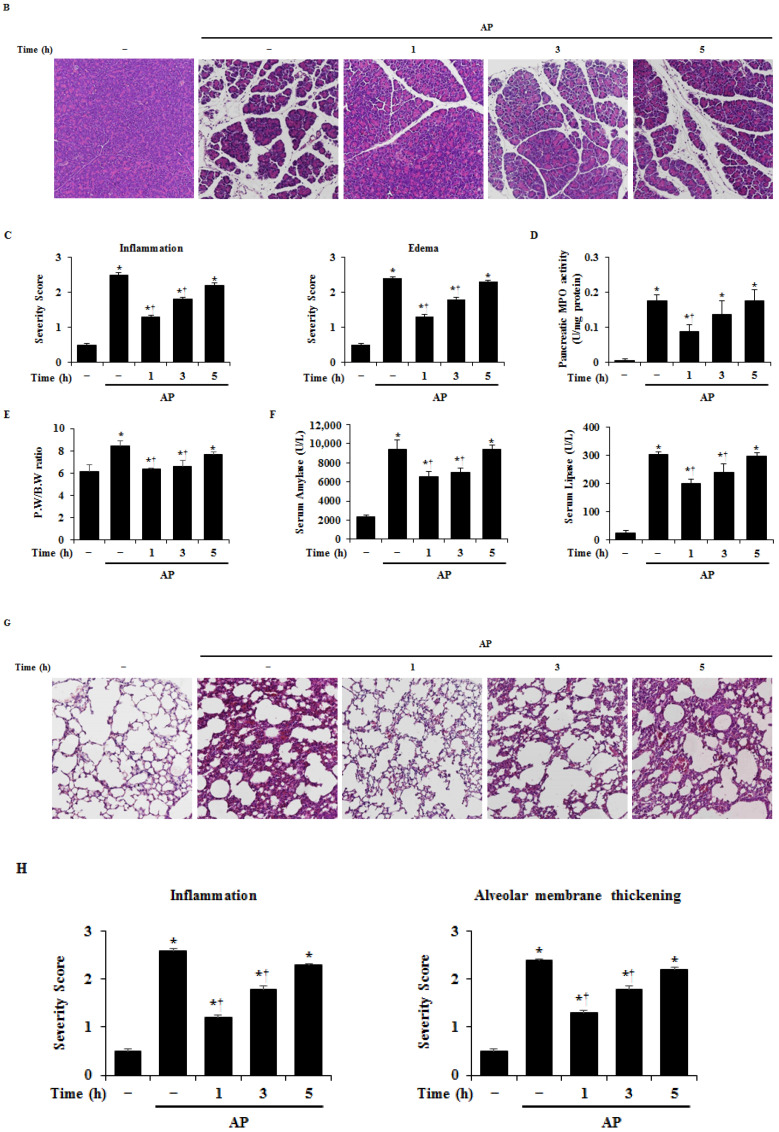
Therapeutic effects of betulinic acid (BA) in cerulein-induced acute pancreatitis (AP). (**A**) BA (10 mg/kg) or dimethyl sulfoxide (DMSO) was administered at 1, 3, or 5 h after the first cerulein injection. Mice were sacrificed at 6 h after the last cerulein injection. Representative hematoxylin and eosin (H&E)-stained (**B**) pancreas and (**G**) lung tissue section (200× magnification). (**C**) Histological sections of the pancreas were scored from 0 (normal) to 3 (severe) for inflammation and edema. (**D**) Pancreatic myeloperoxidase (MPO) activity. (**E**) Pancreas weight/body weight (PW/BW) ratio. (**F**) Levels of serum amylase and lipase. (**H**) Histological sections of lung tissue were scored from 0 (normal) to 3 (severe) for inflammation and alveolar membrane thickening. Data are represented as mean ± SEM for six mice in each group. Results are representative of three experiments. * *p* < 0.05 vs. DMSO treatment alone. ^†^ *p* < 0.05 vs. cerulein treatment alone.

Based on the prophylactic effects of BA on AP, we examined whether BA has a therapeutic effect on AP and pancreatitis-associated lung injury. BA (10 mg/kg) was administered at 1, 3, or 5 h after the first cerulein injection. As shown in Figure 6, under post-treatment with BA at 1 or 3 h, but not at 5 h, there was a significant reduction in the severity of pancreatitis and its associated lung injury.

## 3. Discussion

In this study, the data showed that BA attenuated the severity of AP, as indicated by the reduced levels of amylase and lipase, MPO activity, inflammatory cytokines, and pancreatitis-associated lung injury. In addition, BA administration inhibited NF-κB activation against cerulein challenge, both in vivo and in vitro. These findings suggest that BA attenuates AP by inhibiting NF-κB activation.

AP has increasingly become one of the most prominent acute gastrointestinal disorders throughout much of the world, including Europe, Asia, and North America [31,32,33]. The incidence of AP is increasing, in addition to the burden on medical services [34]. Therefore, there is a need to develop an effective treatment for AP that has no side effects and is low-cost. Betulin has a wide range of biological activities, and is easily converted into BA, which has typically shown to be more potent than betulin. However, its low solubility and oral bioavailability have thus far prevented its additional development [35,36,37]. BA has been reported to have a number of anti-inflammatory effects [38,39,40,41], and being a natural compound, has a low toxicity and is cost-effective [42]. A dose of BA as high as 500 mg/kg is generally recognized as safe in athymic mice, suggesting that it may be an effective option as a new therapeutic agent for AP [43]. The initial injury that causes AP results in acinar cell death, and is accompanied by local pancreatic inflammation, with the recruitment of the inflammatory response [44]. In addition, AP is a complex pathophysiological process involving multiple factors, such as the activation of granulocytes and inflammatory mediators [45,46]. Thus, the regulation of acinar cell damage, pancreatic digestive enzymes, such as amylase and lipase, and inflammatory cell infiltration is important for the treatment of AP. Moreover, lung injury, a common secondary complication of AP, is associated with systemic inflammation and contributes to the majority of AP-associated deaths [47,48]. In this study, BA significantly inhibited pancreatic and lung damage, serum amylase and lipase activity, and MPO activity, suggesting that BA can be used as a precautionary and therapeutic agent for AP (Figure 1, Figure 2 and Figure 6).

During the early stages of AP, proinflammatory cytokines from acinar cells increase leukocyte activation, and trigger an excessive inflammatory response [49,50,51,52]. The inhibition of proinflammatory cytokine production is one of the alleviative methods for the severity of AP [1,53]. Macrophages and neutrophils are the dominant drivers of premature protease activation and acinar cell necrosis [54]. Depletion of macrophages and neutrophils attenuates zymogen activation and pancreatic damage [55,56]. In this study, BA inhibited the production of proinflammatory cytokines such as IL-1β, IL-6, and TNF-α in the pancreas and acinar cells (Figure 3), and reduced the levels of CCL2 and CXCL2 (Figure 4A), suggesting that the inhibition of proinflammatory cytokine production by BA may regulate the expression levels of CCL2 and CXCL2 in the pancreas. CCL2 is a small cytokine that belongs to the CC chemokine family, which recruits monocytes such as macrophages, neutrophils, T cells, and dendritic cells [57,58]. CXCL2 is a small cytokine belonging to the CXC chemokine family, which has a powerful neutrophil chemoattractant [59,60]. Our results showed that BA reduced infiltration of macrophages and neutrophils into the pancreas (Figure 4B,C), suggesting that the reduced infiltration of macrophages and neutrophils into the pancreas may be associated with the reduction of expression levels of CCL2 and CXCL2 by BA. Taken together, our results suggest that BA may regulate the severity of AP and AP-associated pulmonary complications, through the inhibition of the inflammatory response. In recent studies [61,62], the hydroxamate of BA (BHA), a hypoxia mimetic derivative of BA, was used to improve the efficacy of BA against fibrosis associate with inflammatory bowel disease (IBD). BHA provides protection for inflammation and fibrosis in IBD, decreasing the number of macrophages and lymphocytes. Therefore, further study is needed to investigate the effects of BA/BHA on the inflammatory response and fibrosis in AP and chronic pancreatitis.

In the early stages of AP, NF-κB is rapidly activated and triggers several inflammatory responses [63]. The NF-κB pathway plays a central role in regulating the synthesis and transcription of inflammatory cytokines during AP [64]. Pharmacological inhibitors of NF-κB have been shown to attenuate the severity of AP [65]. In NF-κB-deficient mice, the regulation of NF-κB protects against inflammation and tissue damage in pancreatitis [66]. BA has also been shown to regulate NF-κB signaling in previous studies [67,68]. Therefore, we examined the NF-κB pathway in the pancreas and PACs after cerulein treatment. BA treatment inhibited the phosphorylation of IKK and Iκ-Bα, Iκ-Bα degradation, and NF-κB binding activity in the pancreas during AP (Figure 5A–F). NF-κB translocation into the nucleus mostly occurs in PACs during AP [69]. Next, we examined the NF-κB p65 translocation and binding activity in PACs. Pretreatment with BA inhibited NF-κB translocation into the nucleus and NF-κB binding activity (Figure 5G–I). Taken together, our results suggest that BA reduces inflammatory responses in the development of AP by regulating the NF-κB signaling pathway.

## 4. Materials and Methods

### 4.1. Materials

BA, dimethyl sulfoxide (DMSO), hexadecyltrimethylammonium bromide, hematoxylin, eosin, xylene, ethanol, NaCl, KCl, CaCl_2_, MgCl_2_, glucose, and HEPES were purchased from Sigma-Aldrich (St. Louis, MO, USA). Cerulein was purchased from Bachem AG Laboratories (Bubendorf, Switzerland). The Easy-Blue™ Total RNA Extraction Kit and radioimmunoprecipitation assay (RIPA) buffer were purchased from iNtRON Biotechnology (Seongnam, KyungKiDo, Korea). Antibodies against phospho-specific IKKα/β (#2697), phospho-specific Iκ-Bα (#2859), P38 (#9211), JNK (#9251), ERK (#9101), and Iκ-Bα (#9242), as well as total MAPK antibodies against P38 (#9212), JNK (#9252), and ERK (#9102) were purchased from Cell Signaling Technology (Beverly, MA, USA). Antibodies against IL-1β (sc-7884), NF-κB p65 (sc-372), COX-2 (sc-166475), and β-Actin (sc-47778) were purchased from Santa Cruz Biotechnology (Santa Cruz, CA, USA). TNF-α (ab65579) was purchased from Abcam (Cambridge, UK). IL-6 (#11-7061-82) was purchased from Invitrogen; Thermo Fisher Scientific (Waltham, MA, USA).

### 4.2. Animal Models

Female C57BL/6 mice (age: 6 weeks, weight: 15–20 g) were purchased from Samtako BioKorea Co. Ltd. (Osan, KyungKiDo, Korea). All animals were bred and housed in standard shoebox cages in a climate-controlled room with an ambient temperature of 23 ± 2 °C and a 12 h light/12 h dark cycle. All mice were fed standard laboratory chow, allowed water ad libitum under standard conditions with air filtration, and allowed to acclimatize to the new environment for 7 days before induction of pancreatitis. The mice were randomly divided into control and experimental groups.

### 4.3. Experimental Design

AP was induced by intraperitoneal (ip) injection of cerulein (50 µg/kg, *n* = 6 per group for three experiments, total = 18) every hour for 6 consecutive hours. DMSO was administered to the control group under the same conditions. In the pretreatment groups, BA (1, 5, or 10 mg/kg, *n* = 6 per group for three experiments, total = 18) or DMSO (control group, *n* = 6 per group for three experiments, total = 18) was administered by means of ip injection, 1 h before the first cerulein injection. In the post-treatment groups, BA (10 mg/kg, *n* = 6 per group for three experiments, total = 18) or DMSO (control group, *n* = 6 per group for three experiments, total = 18) was administered at 1, 3, and 5 h after the first cerulein injection. The mice were sacrificed at 6 h after the last injection of cerulein, after which the pancreas, lungs, and blood samples were immediately collected for further examination. All experiments were performed three times independently. 

### 4.4. Biochemical Detections

To measure biochemical toxicity, the mice were divided into six groups. Mice received an ip injection of BA (0.1, 1, 5, 10, or 20 mg/kg) or DMSO and were then sacrificed at 24 h after the treatment. The serum was separated by means of centrifugation at 5000 rpm for 5 min. Fresh serum was used for the measurement of alkaline phosphatase, alanine transaminase (ALT), aspartate transaminase, blood urea nitrogen, and creatinine levels using LABOPSECT 008AS (SP) (Hitachi, Tokyo, Japan).

### 4.5. Histological Examination

Pancreatic and lung tissue samples were fixed in 4% paraformaldehyde for 24 h, embedded in paraffin blocks, and cut into 4 μm sections. Tissue sections were stained with hematoxylin and eosin for histological examination under a light microscope. The pancreas and lung sections representing a minimum of 100 fields from each group were randomly semi-quantitatively assessed. As previously reported [23], the pancreas was scored for inflammation (infiltrated inflammatory cells) and edema (separation of acinar cells). The lungs were scored for inflammation (infiltrated inflammatory cells) and thickness (alveolar wall thickness), as previously described [70,71]. Each test was scored on a scale from 0 (normal) to 3 (severe). All experiments were performed three times.

### 4.6. Myeloperoxidase Activity

The sequestration of neutrophils from the pancreas was assessed by measuring myeloperoxidase (MPO) activity in the tissues. The MPO assay was modified according to a previously described method [72].

### 4.7. Pancreatic Edema Analysis

The ratio of pancreatic weight (PW) to body weight (BW) was evaluated as an estimate of the degree of pancreatic edema. At the time of sacrifice, the pancreas and the body of the mice were weighed. PW was divided by BW and multiplied by 1000 to convert the ratio to a natural number.

### 4.8. Determination of Serum Amylase and Lipase Levels

Serum samples for the determination of amylase and lipase levels were obtained at 6 h after the final cerulein injection. Mice were sacrificed via CO_2_ asphyxiation followed by cervical dislocation, after which blood samples were withdrawn from their hearts. Serum amylase and lipase levels were measured using LabOPSECT 008AS (SP).

### 4.9. Reverse Transcription-Quantitative Polymerase Chain Reaction

mRNA transcript levels were analyzed using reverse transcription-quantitative polymerase chain reaction (RT-qPCR) in pancreatic tissues and PACs. Total RNA was isolated using the Easy-Blue™ Total RNA Extraction Kit according to the manufacturer’s instructions. Reverse transcription of RNA to cDNA was performed using a ReverTra Ace^®^ qPCR RT Kit (Toyobo, Osaka, Japan), and qPCR was performed using TaqMan^®^ and THUNDERBIRD™ Probe qPCR Mix, according to the manufacturer’s instructions (Toyobo, Osaka, Japan). For each sample, triplicate test reactions and a control reaction without reverse transcriptase were analyzed for the expression of the gene of interest, and to control for variations in the reactions. qPCR data were normalized to the levels of the housekeeping gene hypoxanthine-guanine phosphoribosyltransferase (HPRT). qPCR was performed at 50 °C for 2 min and 95 °C for 10 min, followed by 60 cycles of amplification at 95 °C for 10 s and 60 °C for 30 s. Forward, reverse, and probe oligonucleotide primers for multiplex real-time TaqMan^®^ PCR were purchased from ABI (Applied Biosystems, Waltham, MA, USA), HPRT (Mm03024075_m1), IL-1β (Mm00434228_m1), IL-6 (Mm00446191_m1), TNF-α (Mm00443258_m1), CCL2 (Mm00441242_m1) and CXCL2 (Mm00436450_m1). The data were analyzed using CFX Maestro™ software (version 2.0; Bio-Rad Laboratories, Hercules, CA, USA). The 2^−ΔΔCq^ method was used to determine relative mRNA expression levels [73]. The expression of COX-2 and GAPDH mRNAs were determined using a ABI Step one Plus (Applied Biosystems, Waltham, MA, USA) with a SYBR Premix Ex Taq (Applied Biosystems, Waltham, MA, USA). The amplification conditions included: 30 s at 95 °C; 50 cycles at 95 °C for 5 s and 60 °C for 60 s each; dissociation for 15 s at 95 °C and 30 s at 60 °C; and then 15 s at 95 °C on a T ABI Step one Plus. The primers for COX-2 and GAPDH were as follows; COX-2 (forward) 5′-AGGAGACATCCTGATCCTGGT-3′ and (reverse) 5′-GTTCAGCCTGGCAAGTCTTT-3′. GAPDH (forward) 5′-TGTGTCCGTCGTGGATCTGA-3′ and (reverse) 5′-TTGCTGTTGAAGTCGCAGGAG-3′. Stepone software (Applied Biosystems, Waltham, MA, USA) was used for data analysis. The Ct values (cycle threshold) were calculated using the crossing point method, and the gene expression levels were measured by a comparison with a standard curve. The expression levels of target genes were normalized to those of GAPDH.

### 4.10. Enzyme-Linked Immunosorbent Assay (ELISA)

Protein levels of IL-1β, IL-6, and TNF-α in the pancreas tissue were analyzed in duplicate in 96-well plates coated with anti-mouse IL-1β, IL-6, and TNF-α monoclonal antibodies in PBS, with an overnight incubation at 4 °C. The plates were washed with PBST and blocked with PBS containing 10% FBS for 2 h at RT. After washing, standards and samples were added and incubated for 3 h at RT. The wells were washed, and biotinylated anti-mouse IL-1β, IL-6, and TNF-α were added and incubated at RT for 1 h. The wells were washed again, avidin-peroxidase was added, and plates were incubated for 30 min at RT. Then, the TMB substrate was added. Color development was measured at 450 nm using an automated microplate ELISA reader. Standard samples were obtained for each sample, using serial dilutions of recombinant IL-1β, IL-6, and TNF-α.

### 4.11. Immunofluorescence

Immunofluorescence was employed to assess IL-1β, IL-6, TNF-α, CD11b, Ly6G, phospho-specific IKKα/β, phospho-specific Iκ-Bα, and Iκ-Bα expression in pancreatic tissues, and NF-κB p65 expression in PACs. The pancreatic tissues were embedded in an optimum cutting temperature medium and then frozen at −80 °C until sectioning. The tissue was cut into 9 μm sections and fixed in 100% methanol at −20 °C for 5 min. The slides were incubated with 3% hydrogen peroxide for 10 min at RT, to block endogenous peroxidase, and then permeabilized with 0.1% Triton™ X-100 for 10 min at RT. The slides were then blocked with 3% bovine serum albumin (BSA) for 1 h at RT, and incubated overnight at 4 °C with the primary antibodies against IL-1β, IL-6, TNF-α, anti-mouse PE conjugated CD11b, anti-mouse FITC conjugated Ly6G, phospho-specific Iκ-Bα, Iκ-Bα and NF-κB p65 (used at a dilution of 1:250 in 3% BSA), and phospho-specific IKKα/β (used at a dilution of 1:100 in 3% BSA). Subsequently, the slides were washed and incubated with the secondary antibody, Alexa Fluor^®^ 568 goat anti-rabbit IgG (used at a dilution of 1:2000 in 3% BSA), for 2 h at RT. The nuclei were then counterstained with DAPI (used at a dilution of 1:2000 in 3% BSA) for 5 min at RT. Finally, the slides were mounted with mounting media, cover-slipped, and visualized using a confocal laser microscope (Olympus, Tokyo, Japan).

### 4.12. Isolation of Mouse PACs

PACs were isolated from the pancreas of C57BL/6 mice using collagenase digestion, as previously described [74].

### 4.13. Cell Viability Assay

Acinar cell viability was analyzed using the 3-(4,5-dimethylthiazol-2-yl)-2,5-diphenyl tetrazolium bromide (MTT) assay. Briefly, PACs were pretreated with BA (1, 5, or 10 μM) for 1 h, and then stimulated with cerulein (10 nM) for 6 h. Thereafter, the cells were incubated with MTT (5 mg/mL) solution for 30 min at 37 °C. The suspension was removed, and the formazan crystals formed were dissolved in 200 μL of DMSO. The cells were seeded into 96-well plates in duplicate, and the absorbance was determined using a microplate ELISA reader at 540 nm. The number of viable cells was expressed as a percentage of control cells.

### 4.14. Isolation of Mouse Pancreatic Cells

Mouse pancreatic cells were isolated, as previously described [56], from each group. In brief, the pancreas was rapidly removed then chopped into small pieces in a FACS buffer (Dulbecco’s phosphate-buffer saline containing 10% fetal calf serum and 5 mM EDTA). Suspended tissues were grinded by pipetting for 30 s, and filtered using 100 μm nylon mesh. After filtration, the remaining large pieces were transferred to a fresh FACS buffer, and these steps were repeated once more. All filtered cells were centrifuged (250× *g*, 25 min, 4 °C) and the supernatant was discarded. Remaining cell pellets were suspended in a fresh FACS buffer to stain for flow cytometry experiments.

### 4.15. Flow Cytometry Analysis

Pancreatic cells isolated from each group were washed by a fresh FACS buffer for three times, then cell suspensions were treated with the anti-mouse CD16/CD32 (1:100 ratio, clone 93, eBioscience, Santa Clara, CA, USA) for 15 min to block the Fc receptor. Cell suspensions were subsequently labeled with anti-mouse PE-Cyanine 5 conjugated CD11b (1:500 ratio, clone M1/70, eBioscience, Santa Clara, CA, USA) and anti-mouse FITC conjugated F4/80 (1:500 ratio, clone BM8, eBioscience, Santa Clara, CA, USA), and/or anti-mouse PE conjugated Ly6G (1:500 ratio, clone RB6-8C5, eBioscience, Santa Clara, CA, USA) for 30 min on ice in the dark to detect macrophages or neutrophils. Stained cells were measured with the FACS Calibur (Becton Dickinson, San Jose, CA, USA) and flow cytometric data were analyzed using CellQuestPro software.

### 4.16. Western Blot Assay

Proteins were prepared from pancreatic tissues and PACs in 1× RIPA lysis buffer with a 1% protease inhibitor cocktail and 1% phosphatase inhibitor. The samples were boiled in a sample buffer (62.5 mM Tris-HCl pH 6.8, 2% sodium dodecyl sulfate (SDS), 20% glycerol, and 10% 2-mercaptoethanol). The protein concentrations were determined using the bicinchoninic acid method. Each well was loaded with 20 µg of total protein, and the proteins were separated on a 10% SDS-polyacrylamide gel and transferred to a polyvinylidene diflouride membrane (GE Healthcare Life Sciences, Little Chalfont, Buckinghamshire, UK). The membranes were blocked with 5% skim milk in PBS-Tween 20 (PBST) for 2 h at RT, and incubated with primary antibodies overnight at 4 °C. Primary antibodies were as follows: Iκ-Bα, β-Actin, COX-2, pP38, pJNK, pERK, P38, JNK, and ERK (used at a dilution of 1:1000 in PBST). After washing, the membranes were incubated with horseradish peroxidase (HRP)-conjugated goat anti-rabbit IgG secondary antibodies (used at a dilution of 1:5000 in PBST) and HRP-conjugated goat anti-mouse IgG secondary antibody (used at a dilution of 1:5000 in PBST) for 1 h at RT. Protein bands were developed using chemiluminescence with an EZ-Western Lumi Pico Kit (DoGenBio, Seoul, Korea), according to the manufacturer’s recommended protocol. Protein bands were captured using Ez-capture ST (AE-9160PH, ATTO, Tokyo, Japan), and quantitative analysis was performed by using Image J software (National Institutes of Health, Bethesda, MD, USA).

### 4.17. Nuclear Extraction and NF-κB Binding Activity Determination

NF-κB binding activity was measured in the pancreatic tissues and PACs. Nuclear extracts of tissues and cells were prepared using a nuclear extraction kit (Chemicon, Chungcheng-bukdo, Korea). Nuclear extraction was used to determine the NF-κB binding activity, which was measured using the NF-κB p65 Binding Activity Assay Kit from Active Motif (Carlsbad, CA, USA).

### 4.18. Statistical Analysis

All results were expressed as mean ± standard error of the mean. The significance of differences was determined using a two-way analysis of variance. Differences were considered statistically significant at *p* < 0.05. The results were similar in the three independent experiments.

## 5. Conclusions

In conclusion, we demonstrated that BA attenuates the severity of AP, by inhibiting pancreatic injury and inflammatory responses. Our data suggest that BA is a potential novel strategy for the treatment of AP.

## Figures and Tables

**Table 1:** Biochemical Toxicity.

Group	ALP (IU/L)	ALT (IU/L)	AST (IU/L)	BUN (mg/dL)	CREA (mg/dL)
DMSO	241.50 ± 38.41	29.50 ± 4.95	159.33 ± 29.91	21.25 ± 0.96	0.15 ± 0.03
BA 0.1 mg/kg	242.60 ± 19.32	25.50 ± 3.54	155.67 ± 9.29	19.60 ± 1.34	0.14 ± 0.01
BA 1 mg/kg	236.20 ± 32.91	29.20 ± 5.58	157.00 ± 13.93	20.25 ± 0.50	0.15 ± 0.02
BA 5 mg/kg	240.00 ± 20.77	29.17 ± 7.36	161.20 ± 29.63	19.33 ± 4.16	0.16 ± 0.02
BA 10 mg/kg	234.50 ± 17.25	28.33 ± 0.58	156.00 ± 30.17	17.50 ± 1.00	0.13 ± 0.01
BA 20 mg/kg	237.33 ± 25.48	53.50 ± 7.78 *	156.20 ± 22.38	21.00 ± 4.64	0.16 ± 0.01

ALP, alkaline phosphatase; ALT, alanine aminotransferase; AST, aspartate transaminase; BUN, blood urea nitrogen; CREA, creatinine. Data are represented as means ± SEM for six mice in each group. Results are representative of three experiments. * *p* < 0.05 vs. DMSO treatment alone.

## Data Availability

The datasets used and/or analyzed during the current study are available from the corresponding author on reasonable request.

## Supplementary Materials

The following are available online at https://www.mdpi.com/article/10.3390/ijms22136871/s1.
