# Supplementary material for: Betulinic Acid Ameliorates the Severity of Acute Pancreatitis via Inhibition of the NF-κB Signaling Pathway in Mice
Source: Int J Mol Sci. 2021 Jun 26;22(13):6871. doi: 10.3390/ijms22136871 (PMC8268208; doi:10.3390/ijms22136871)
Supplement: Supplementary file 1 [file ijms-22-06871-s001.zip › ijms-1266351-supplementary.pdf]

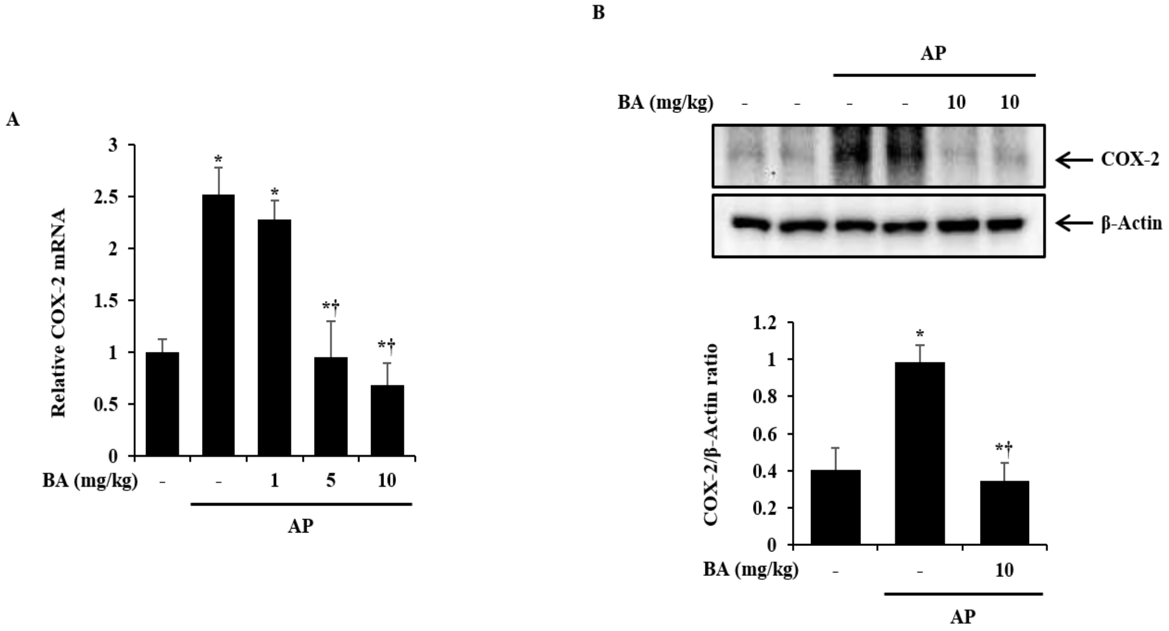


**Figure S1.** Effect of betulinic acid (BA) on expression of cyclooxygenase 2 (COX-2) in cerulein-induced acute pancreatitis (AP). (A) Pancreatic mRNA levels of COX-2 was determined using reverse transcription-quantitative polymerase chain reaction (RT-qPCR). (B) Pancreatic COX-2 protein was measured using western blotting. β-actin was used as the loading control. Western blot of COX-2 was quantified by densitometry and normalized to β-actin. Data have been represented as mean ± SEM for six mice in each group. Results are representative of three experiments. *$P < 0.05$ *vs.* DMSO treatment alone. $^{\dagger}P < 0.05$ *vs.* cerulein treatment alone.

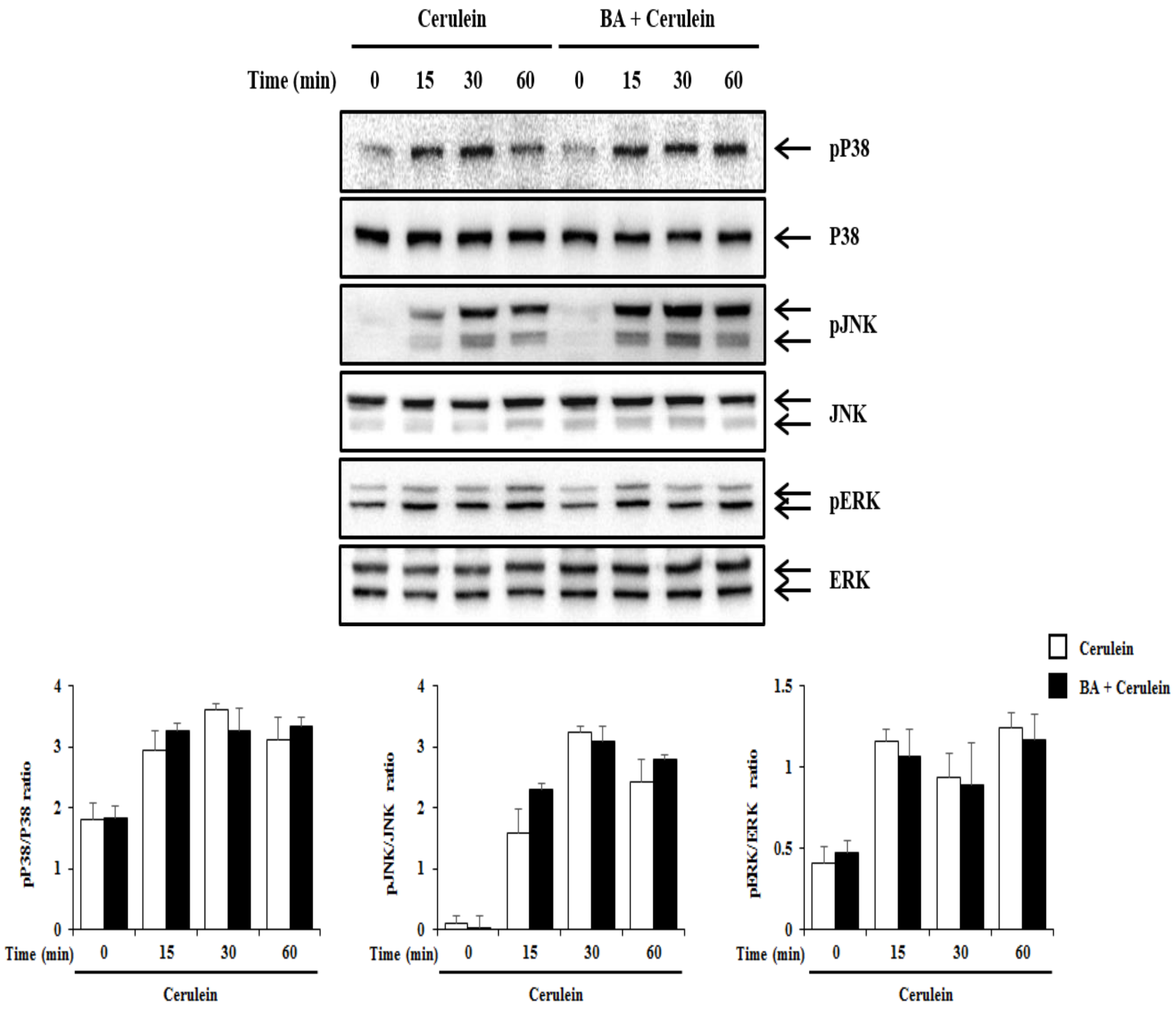


**Figure S2.** Effect of betulinic acid (BA) on phosphorylation of P38, c-Jun *N*-terminal kinase (JNK) and extracellular signal-regulated kinase (ERK) activation. Phosphorylation of P38, JNK and ERK were measured using western blotting. Western blot of phosphorylation of P38, JNK and ERK were quantified by densitometry and normalized to P38, JNK and ERK, respectively. Data have been represented as mean ± SEM for six mice in each group. Results are representative of three experiments.
